# *Anopheles cinereus* implicated as a vector of malaria transmission in the highlands of north-west Ethiopia

**DOI:** 10.1186/s13071-019-3797-9

**Published:** 2019-11-25

**Authors:** Wossenseged Lemma, Kassahune Alemu, Meserete Birhanie, Ligabaw Worku, Julie Niedbalski, Mary Ann McDowell, Neil F. Lobo

**Affiliations:** 1 Department of Medical Parasitology, College of Medicine and Health Sciences, University of Gondar, Gondar, Ethiopia; 2 Tropical Infectious Diseases Research Center, College of Medicine and Health Sciences, University of Gondar, Gondar, Ethiopia; 3 Department of Epidemiology and Biostatics, College of Medicine and Health Sciences, University of Gondar, Gondar, Ethiopia; 4 Department of Biological Sciences, Eck Institute for Global Health, University of Notre Dame, Notre Dame, IN USA

**Keywords:** Malaria, *Anopheles*, Ethiopia, Residual transmission

## Abstract

**Background:**

Transmission of malaria in the highlands of Ethiopia is poorly understood and usually attributed to importation by mobile populations or local transmission by *Anopheles arabiensis*. To characterize and identify *Anopheles* species present in a highland area of northern Ethiopia, adult and larval collections were performed in Gondar town and the neighboring Senbet Debir village (Dembia district, > 2000 meters above sea level, masl), in addition to Bahir Dar town (capital of Amhara region) and Kumer Aftit village (Metema district, < 2000 masl).

**Methods:**

CDC-light traps were used to collect adult mosquitoes and larval collections were performed from rain pools for rearing into adults for species identification. Collections were made September-March 2016–2018. Adult mosquitoes were identified morphologically and a subset of randomly chosen specimens were identified to species by sequencing the ribosomal DNA internal transcribed spacer region 2 (ITS2) and mitochondrial DNA cytochrome *c* oxidase subunit 1 (*cox*1).

**Results:**

The primary species of *Anopheles* identified at elevations higher than 2000 masl was *An. cinereus*, which was confirmed molecularly by ITS2 and *cox*1 sequencing. Interestingly, two unknown species were also sequenced, in addition to two specimens of *An. pretoriensis*. The species collected at sites with elevations less than 2000 masl (Bahir Dar town and Kumer Aftit village) was *An. arabiensis*. Three *Plasmodium falciparum*-positive specimens were identified molecularly as *An. cinereus*.

**Conclusions:**

The presence of *Plasmodium*-positive *An. cinereus* in areas greater than 2000 masl incriminates this species as a potential vector contributing to non-peak malaria transmission in Ethiopian highland areas.

## Background

With the call to eliminate malaria, there is a need to examine more atypical transmission zones. Highland areas (> 1500 meters above mean sea level, masl) are usually considered to be malaria free due to the inability of the vectors to survive and transmit malaria [1]. Traditionally, highland malaria is thought to result from importation of cases from malaria endemic lowland areas through migrant and mobile human populations. However, the East African highlands are now described as having unstable malaria transmission with additional reports of several epidemics [2]. The Ethiopian highlands make up 60% of all highlands in East Africa [3] and approximately 43% of Ethiopia’s total population currently lives in highland areas at altitudes ranging from 1600 to 2400 masl [4].

A study investigating malaria risk factors in malaria endemic kebeles (sub-district) in Gondar town in 2004, indicated that the proximity of households to larval sites (rivers) during the December dry season increased the likelihood of being malaria positive by 2.43 times - assuming all other factors being constant [5]. Similarly, in Adama, people in households within 250 meters of a flood plain have been demonstrated to have a 22-fold higher risk of contracting malaria than households more than 950 meters away [6]. Though both studies lack entomological investigations, data from other sites demonstrate that proximity to larval sites increases risk [7]; this may be true in Gondar town with river-edge based *Anopheles* larval sites possibly driving transmission.

Cyclic malaria epidemics in Ethiopia highlands were believed to be associated with the unusually high temperature and rainfall related to *El Niño*-based weather patterns [8]. Similarly, a rise in temperature due to climate change was reported to increase annual malaria incidence in Ethiopia, presumably due to new territorial expansion of vectors to highland areas [4]. Therefore, entomological evidence demonstrating movement of malaria vectors to highland areas is required.

The Ethiopian highlands of Amhara, that extend into the Tigray zone and into Eritrea, have similar *Anopheles* mosquito fauna including *An. arabiensis*, *An. pharoensis*, *An. christyi*, *An. cinereus*, *An. turkhudi*, *An. squamosus*, *An. garnhami*, *An. coustani*, *An. ziemanni*, *An. demeilloni*, *An. dʼthali*, *An. funestus*, *An. rhodesiensis* and *An. macmahoni* [9]. In Ethiopia, *An. arabiensis* was reported as the primary vector, while *An. pharoensis*, *An. funestus* and *An. nili* play a secondary role [10, 11]. The identification of a known malaria vector, *An. arabiensis,* at elevations of 1720 to 1921 masl in Kenya, indicates the possibility of local malaria transmission on the Mount Kenya highlands [12]. Multiple vectors including species previously unknown to vector *Plasmodium* and unidentified species were also identified in the highlands in western Kenya (up to 1700 masl) [13, 14]. These studies indicate that there may be vectors present and able to transmit malaria in unexpectedly high elevations. Although vector incrimination studies have not been conducted in Ethiopian highland areas, the presence of the documented vector (*An. cinereus*) in areas greater than 2000 masl in Eritrea positive for *Plasmodium* sporozoites [15] indicates a role for this species in malaria transmission.

Here we investigated possible malaria vectors in the Amhara region, Ethiopia. Eighty percent of the Amhara region is malarious with ~ 75% of ~ 20 million people being at risk of contracting malaria [16]. Lowland agricultural areas within Amhara are highly endemic for malaria. Highland areas surrounding Lake Tana in Fogera, Gondar Zuria and Dembia districts, including Bahir Dar (the capital city of Amhara region) and Gondar town, are recognized as high-risk malaria areas, especially in previous years when effective drugs were not available.

## Methods

**Fig. 1:**
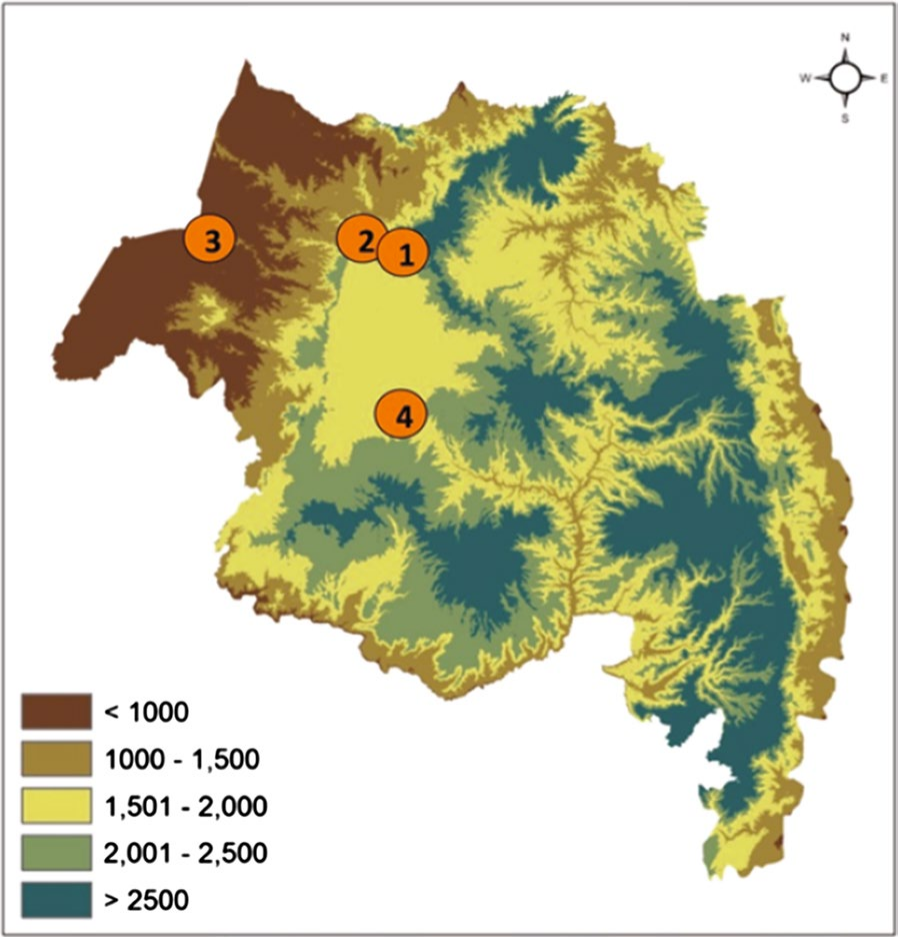
Elevation map of Amhara region, Ethiopia, depicting the 4 sites: Site 1, Gondar; Site 2, Senbet Debir; Site 3, Kumer Aftit; and Site 4, Bahir Dar

CDC-light traps (LTs), were set inside human habitation, mixed habitation and outdoor in cattle sheds to collect mosquitoes in two sites: Gondar (Kaha, Azezo, Bamba Sefer) during October-January 2016/2017 and Senbet Debir (a village near Ayimba town, Dembia district) during March 2018 (Fig. 1), for a total of 223 trap nights. Larval collections were performed from rain pools in Kumer Aftit village, and Bahir Dar town over the wet season (September-November) in 2016–2017. *Anopheles* adult females from either the adult collections or those reared from immature stages were identified to species using the appropriate key [17]. A subset of randomly chosen morphologically identified specimens were identified to species by sequencing the ribosomal DNA internal transcribed spacer region 2 (ITS2) and mitochondrial DNA cytochrome *c* oxidase subunit 1 (*cox*1) (*n* = 32) loci [13, 18]. In short, ITS2 and *cox*1 sequences generated from sequencing PCR products from individuals were compared and grouped into ‘species’ groups (contigs) based on single nucleotide polymorphisms and deletions/insertions using a sequence similarity threshold using a threshold of 98% for ITS2 and 95% for *cox*1. Following manual inspection, consensus contigs were compared (BLASTn) to the NCBI nr database for species identification. DNA was extracted from the head and thorax of *Anopheles* specimens and used to amplify the *Plasmodium-*specific *cox*1 using a single step conventional PCR [19]. PCR products indicating the presence of *Plasmodium* in the mosquito specimens were sequenced to identify the species of *Plasmodium* present.

## Results and discussion

Of the 1221 adult *Anopheles* females captured over 213 trapping nights in Gondar and Senbet Debir, six species were identified morphologically: *An. cinereus*, *An. pharoensis*, *An. wilsonii*, *An. funestus*, *An. demeilloni* and *An. coustani* (Table 1).

**Table 1:** Morphological identification of *Anopheles* females captured in Gondar and Senbet Debir using CDC-LTs and human, cow and human + cow baits

Site	*An. cinereus*	*An. pharoensis*	*An. wilsonii*	*An. funestus*	*An. demeilloni*	*An. coustani*
Gondar	951	2	1	28	6	4
Senbet Debir	225	0	0	4	0	0
Total	1176	2	1	32	6	4

The use of sequencing two specific ‛species-indicatingʼ regions (ITS2 and *cox*1) enabled the detection of two novel sequences indicating the presence of two possible novel species which would have escaped detection in the presence of only morphological identification. The importance of using molecular techniques to complement morphological identifications was validated with the documentation of two possible new species. ITS2 and *cox*1 sequencing of 380 randomly chosen samples (across sites, trapping periods and trapping types) demonstrated the presence of four species. The primary *Anopheles* identified was *An. cinereus* (*n* = 325) consisting of 90% of the samples in Gondar and 76% of the samples in Senbet Debir. An unknown species, misidentified as both *An. cinereus* and *An. funestus*, was closest to a similar Kenya highlands species [13, 14] (though not identical) and was called *Anopheles* sp. 1 BSL-2014-cf1 (*n* = 52). This species consisted of 9.5% of the collection in Gondar and 22% of the collection in Senbet Debir (Table 2). Two specimens of *An. pretoriensis* and a single sample of the unknown *Anopheles* sp. KHH11 were found in Senbet Debir and Gondar, respectively. Larval collections in the lower elevation sites of Kumer Aftit and Bahir Dar consisted of only *An. arabiensis*. These larvae were commonly found in rain created tiny pools during September-November main malaria season. At higher elevation sites (above 2000 masl), where *An. arabiensis* was absent from collections, the primary species present were *An. cinereus* and the unknown *Anopheles* sp. 1 BSL-2014 - cf1.

**Table 2:** A comparison of morphological and molecular identifications

Site	Morphological identification	Molecular identification				
		*An. arabiensis*	*An. cinereus*	*An. pretoriensis*	*Anopheles* sp. 1 BSL-2014-cf1	*Anopheles* sp. KHH11
Gondar	*An. cinereus*	–	228	0	17	1
	*An. funestus*	–	–	–	7	–
Senbet Debir	*An. cinereus*	–	97	1	26	–
	*An. funestus*	–	–	–	2	–
	*An. ziemanni*	–	–	1	–	–
Kumer Aftit	*An. arabiensis*	94	–	–	–	–
Bahir Dar	*An. arabiensis*	41	–	–	–	–
ITS2 Accession #			MN460356	MN460357	MN460358	MN460359
*cox*1 Accession #			MN453868	MN453869	MN453870	MN453871

Collections in Gondar and Senbet Debir were adults while those in Kumer Aftit and Bahir Dar were adults reared from larval collections. Four species (including two unknown species) were identified

All samples processed molecularly were screened for *Plasmodium* species by PCR. Of the total 325 *An. cinereus* molecularly identified (255 from Gondar and 70 from Senbet Debir village), three (1%) were positive for *P. falciparum*. The other species were negative. All samples that were positive for *Plasmodium* were from indoor (human only, human + cattle bait) CDC-LT collections near the Gabi Kura river in Senbet Debir (March 2018). It is important to note that the *Plasmodium-*positive PCR indicates the presence of *Plasmodium* DNA only, and not infectiousness. Although all samples were dissected (head and partial thorax used), there remains the possibility that the DNA may originate from either infectious sporozoites, or a non-infectious blood meal. In either case, these samples of *An. cinereus*, a species that has been demonstrated to carry sporozoites [15], is either infectious or fed on infected individuals, both suggesting its integration into intervention strategies.

Lower temperatures or a lack of the primary and expected vector, *An. arabiensis*, in highland areas may not be enough to conclude that there is no endemic malaria in the highlands, especially with warming temperatures [12]. The vector status of *An. cinereus* implicated in the neighboring mountainous areas of Eritrea [15] suggests that this species may play an important role in transmission of non-seasonal malaria transmission in highland areas. In addition, the second-most common species found, *Anopheles* sp. 1 BSL-2014 - cf1, is closest to *Anopheles* sp. 1 BSL-2014 found at another highlands site in Kenya [13, 14]. In these previous studies, this new species group was morphologically identified as *An. demeilloni* but placed in the subgenus *Cellia*, Myzomyia series based on sequence similarity [13, 14]. Approximately 1% (3/325) of the *An. cinereus* adults collected in Gondar town and surrounding Senbet Debir mountainous areas were found to be positive with *Plasmodium*. The existence of this species in transmission relevant times and spaces may explain the residual transmission occurring in Gondar town and surrounding highland areas, especially during the dry season, when progressive decline in volume and speed of rivers creates ideal tiny pool habitats for the larvae of *An. cinereus*.

## Conclusions

Over the past decade, Ethiopia has been working on a malaria elimination program to control malaria; however, atypical low level transmission still occurs in the dry season and in high altitude regions, areas that are usually not targeted for intervention strategies. The development of focal intervention strategies that are based on and cater to local transmission dynamics is vital to the success of the call to regional malaria elimination towards global eradication. The characterization of the entomological drivers of transmission in relationship to epidemiological data is the first step in the development of a baseline understanding of transmission dynamics, and hence an intervention strategy. Identification of *Plasmodium-*positive *An. cinereus* warrants further studies on the role of this species in malaria transmission in the highland areas of Ethiopia.

## Data Availability

The data supporting the conclusions of this article are included within the article. Consensus sequences for ITS2 and *cox*1 were submitted to the GenBank database under the accession numbers MN460356-MN460359 and MN453868-MN453871, respectively. Individual sequences are available upon request.

## Abbreviations

CDC-LT: Center for Disease Control light traps
*cox*1: cytochrome *c* oxidase subunit 1 gene
DNA: deoxyribonucleic acid
ITS2: internal transcribed spacer region 2
masl: meters above sea level
PCR: polymerase chain reaction
